# Spatiotemporal expression pattern of ceramide kinase-like in the mouse retina

**Published:** 2010-12-03

**Authors:** Sharon Vekslin, Tamar Ben-Yosef

**Affiliations:** Department of Genetics and The Rappaport Family Institute for Research in the Medical Sciences, Rappaport Faculty of Medicine, Technion-Israel Institute of Technology, Haifa, Israel

## Abstract

**Purpose:**

The *CERKL* gene encodes for ceramide kinase-like, a novel protein of unknown function. *CERKL* mutations are associated with a severe retinal phenotype. The purpose of this work was to investigate alternative splicing, and the temporal and spatial expression pattern of CERKL in the mouse retina.

**Methods:**

Reverse Transcription-Polymerase Chain Reaction (RT–PCR) analysis of mouse retina RNA was used to study the expression of *Cerkl* at various developmental time points, and to identify its various splice-isoforms. A specific anti-CERKL antibody was developed and used for immunostaining to study the localization of the endogenous CERKL protein in retina-derived cell lines and in the mouse retina.

**Results:**

*Cerkl* is expressed in the mouse eye as early as embryonic day 14. A total of seven different *Cerkl* splice-isoforms were identified in the mouse retina. The subcellular localization of CERKL in retina-derived cell lines is variable: CERKL is diffusely distributed in the cytoplasm, and in many cells, it is highly concentrated in the perinuclear region. In most, but not all cells, CERKL is also highly concentrated in the nucleus. In the mouse retina, CERKL is located in the ganglion cell layer, in amacrine cells of the inner nuclear layer, and in photoreceptors. CERKL is highly expressed in cone photoreceptors; however, its expression level in rod photoreceptors is very low. In cultured cells, CERKL is detected in the nucleus, but in retinal cells in situ, it is mostly located in the cytoplasm.

**Conclusions:**

The expression of *Cerkl* in both mature and embryonic mouse retina and the severe retinal phenotype associated with human *CERKL* mutations indicate that this gene plays a crucial role in retinal activity, and that it may be important for retinal development as well. The high expression level of CERKL in cones correlates with the CERKL-associated phenotype in humans. Whether nucleocytoplasmic transport of CERKL actually occurs in vivo under certain conditions and its functional significance remain to be discovered.

## Introduction

Hereditary retinal degeneration (HRD) is a clinically and genetically heterogeneous group of diseases that cause visual loss due to progressive loss of rod and/or cone photoreceptor cells in the retina [1]. In cone-rod degeneration (CRD), cone involvement initially exceeds that of rods, and thus, reduced visual acuity, photophobia, and defective color vision are prominent early symptoms [2]. Rod photoreceptor degenerations involve a secondary loss of cones and are called retinitis pigmentosa (RP). RP classically manifests with night blindness and progressive peripheral visual field loss [3].

The RP26 locus was originally mapped to human chromosome 2q31-q33 in a large Spanish family [4]. The underlying gene was identified in 2004 and named ceramide kinase-like (*CERKL*) [5]. To date, six *CERKL* mutations have been reported [5–9]. Although the *CERKL*-associated phenotype was originally characterized as RP [4,5], it is now clear that mutations in *CERKL* are responsible for a distinct form of HRD, characterized by early macular involvement with roughly parallel cone and rod loss, resulting in a deficit in both peripheral and central vision. This phenotype is diagnosed in some patients as CRD [6–10].

The peptidic sequence of the main human CERKL variant encodes for a protein of 532 amino acids (AA), with a molecular weight of approximately 58 kDa. CERKL is a homolog of the ceramide kinase (CERK) protein, and both proteins harbor a kinase domain related to the diacylglycerol kinases (DAGK) and a pleckstrin homology (PH) domain. In addition, a CERK-specific region that is downstream from the catalytic core and bears a putative Ca^2+^/calmodulin binding motif is also present in CERKL [11,12]. Several studies have been conducted to prove biochemical similarity between CERK and CERKL enzymatic activities. However, so far there has been no evidence that CERKL phosphorylates ceramide or any other lipid substrate in vitro [11,13] or in vivo [14].

*CERKL* transcripts are alternatively spliced in the human retina. Reverse Transcription-Polymerase Chain Reaction (RT–PCR) analysis of human retina mRNA led to the identification of six spliced transcripts that resulted in several protein isoforms [13]. Due to the lack of a specific antibody, the expression pattern of *Cerkl* in the mouse retina was studied using in situ hybridization. *Cerkl* was shown to be expressed in the ganglion cell layer (GCL), and to a lesser extent in the inner nuclear layer (INL) and the photoreceptor cell layer (PRL) [5]. The subcellular localization of CERKL was studied using transfection of the full-length cDNA fused to either green fluorescent protein (GFP) [11,12,15] or haemagglutinin (HA)-tag [13] into COS-1 [11,12] or COS-7 [13,15] cells. The tagged CERKL protein was found in many cellular compartments, including the cytoplasm, perinuclear region, and nucleus; within the nucleus CERKL was abundantly found in nucleoli. Interestingly, there was intercellular variability in the expression pattern: CERKL was present in the nucleus of some, but not all cells within the same culture [11–13,15]. Two putative nuclear localization signals (NLS) and two putative nuclear export signals (NES) were identified in the CERKL protein. Mutagenesis and deletion experiments indicated that these sequences are involved in CERKL nuclear import and export in cultured cells [12,15].

In summary, *CERKL* is a novel gene of unknown function. The severe retinal phenotype associated with human *CERKL* mutations indicates that *CERKL* plays a crucial role in retinal activity. To further study the role of CERKL in the normal retina and in pathologic conditions, we developed a specific anti-CERKL antibody and used it to study the localization of the endogenous CERKL protein in retina-derived cell lines and in the mouse retina.

## Methods

### Animal use

C57BL/6 mice were obtained from Harlan Laboratories Inc. (Jerusalem, Israel). Animal care guidelines comparable to those published by the Institute for Laboratory Animal Research (Guide for the Care and Use of Laboratory Animals) were followed and the research was approved by the Committee for the Supervision of Animal Experiments, Technion - Israel Institute of Technology.

### RNA analysis

Total RNA was isolated from mouse tissues using Tri reagent (Sigma-Aldrich, St. Louis, MO). For expression analysis, reverse transcription (RT) was performed with 1 µg of total RNA in a 20 µl reaction volume using Superscript II reverse transcriptase (Invitrogen, Carlsbad, CA) with random primers. PCR was performed with 1 µl of cDNA in a 25 µl reaction volume in the presence of 5× Readymix (LAROVA GmbH, Teltow, Germany) and 10 pmol of each forward and reverse primer. Primer sequences were as follows: mouse *Cerk*, 5′-GTT CAG CGA GGT GCT GCA TG-3′ and 5′-CTG CCT GCA CAC GAA GCA CC-3′; mouse *Cerkl*, 5′-ATG TTC CGG GGG AGA CGC AGG AG-3′ (located in exon 1) and 5′-CGT GGA GTC CTT TAG TTT GTT CCG-3′ (located in exon 2); and *β-actin*, 5′-GTC CAC ACC CGC CAC CAG TTC-3′ and 5′-CCA GAG GCA TAC AGG GAC AGC-3′.

For the identification of *Cerkl* splice-variants, RT–PCR was performed with the AccuScript High Fidelity RT–PCR system (Stratagene, La Jolla, CA). RT priming was performed with oligo dT (Stratagene). PCR was performed with primers derived from *Cerkl* exons 1 and 14 (5′-ATG TTC CGG GGG AGA CGC AGG AG-3′ and 5′-CAC AAC CAC TCA CAG GAG AAC-3′). PCR products were gel-purified and cloned into the pGEM-Teasy plasmid vector (Promega, Madison, WI). Plasmids derived from 21 independent clones were digested separately by DdeI and HphI endonucleases (New England Biolabs, Beverly, MA) and then underwent electrophoresis on a 2% agarose gel. Clones demonstrating unique digestion patterns were sequenced and analyzed.

### Cell culture

Cells were grown at 37 °C and 5% CO_2_. ARPE-19 cells (American Type Culture Collection (ATCC), Manassas, VA) were grown in DMEM:F12 (1:1) medium (Biologic Industries, Beit Ha’emek, Israel) containing 10% fetal bovine serum (FBS), 1% penicillin/streptomycin, and 2.5 mM L-glutamine; 661W cells (obtained from Dr. Muayyad Al-Ubaidi, The University of Oklahoma) were grown in high-glucose (4.5 g/l) DMEM medium containing 2.5 mM L-glutamine, 110 mg/l sodium pyruvate, 20% FBS, 1% penicillin/streptomycin (Biologic Industries), 40 µg/l hydrocortisone 21-hemisuccinate, 40 µg/l progesterone, 32 mg/l putrescine, and 0.004% β-mercaptoethanol (Sigma-Aldrich).

### Generation of an antibody against CERKL

For the generation of the anti-CERKL antibody (named RA), we synthesized two short peptides (15 AA each) located in specific mouse CERKL regions (peptide 1: AA 109–123; peptide 2: AA 485–499). Each peptide was linked to KLH and a mixture of the two peptides was injected together with CFA or IFA adjuvants into rabbits (Sigma-Aldrich, Rehovot, Israel). Rabbits were bled following 5 injections over a period of 3 months. The antibody was affinity-purified for a better specificity in western blot and immunofluorescence analyses.

### Expression of recombinant CERKL isoforms in bacteria

cDNA fragments encoding for CERKL isoforms a’, b’, and d’ were cloned into the pQE-80L vector (Qiagen, Valencia, CA), and transformed into the DH5α bacterial strain. The protein products fused to a 6X-His tag were expressed under IPTG induction and purified using Ni-NTA resin (Qiagen).

### Commercial antibodies

Primary antibodies used were rabbit polyclonal antibody against GAPDH; goat polyclonal antibody against blue opsin (OPN1SW; Santa Cruz Biotechnology, Santa Cruz, CA); and mouse monoclonal antibodies against PAX6 (A. Kawakami, obtained by means of the Developmental Studies Hybridoma Bank, Department of Biology, The University of Iowa, IA), Fibrillarin, and Rhodopsin (Abcam, Cambridge, MA). Secondary antibodies used were Cy3-conjugated donkey antirabbit IgG, Cy2-conjugated donkey antimouse IgG, Cy2-conjugated donkey antirabbit IgG, Cy3-conjugated donkey antimouse IgG, Cy3-conjugated donkey antigoat IgG, peroxidase-conjugated AffiniPure goat-antimouse, and goat-antirabbit IgG (Jackson ImmunoResearch Laboratories, West Grove, PA).

### Immunocytochemistry

Cells were seeded on coverslips in 6-well plates for 24 h, rinsed with phosphate buffered saline (PBS; 140 mM NaCl, 10 mM Na_2_HPO_4_, 2 mM KH_2_PO_4_, 2.7 mM KCl, pH 7.4), and fixed with 4% paraformaldehyde for 20 min at room temperature. After three rinses in 1× PBS, membrane permeabilization in 0.5% Triton X-100 in 1× PBS was performed for 10 min, followed by three rinses in 1× PBS, and 1 h blocking in 5% FBS at room temperature. Cells were then incubated overnight at 4 °C with primary antibodies in the detection solution (3% FBS and 0.1% Triton X-100 in 1× PBS). After rinsing in 1× PBS, cells were incubated with secondary antibodies and TO-PRO-3 (Invitrogen) in the detection solution for 1 h at room temperature. Coverslips were rinsed with 1× PBS and mounted onto slides using Vectashield with or without 4',6-Diamidino-2-phenylindole (DAPI; Vector Laboratories, Burlingame, CA). Images were taken with a Zeiss Axioscop 2 fluorescent upright microscope and with a Bio-Rad Radiance 2000 confocal microscope.

### Immunohistochemistry

Whole eyeballs were fixed in 4% paraformaldehyde at 4 °C, dehydrated in a graded ethanol series, and embedded in paraffin. Dewaxed paraffin sections (8 µm) were examined immunohistochemically as previously described [16]. Nuclear staining was obtained using TO-PRO-3 (Invitrogen). Slides were viewed with a Bio-Rad Radiance 2000 confocal microscope. As a negative control, the RA antibody was pre-absorbed with fivefold gram-mass of the recombinant CERKL protein (isoform a’) or with a non-specific protein (BSA) for 1 h at room temperature.

### Western blot analysis

For total protein extraction, retinas were subjected to 0.5 M HEPES buffer (pH 7.3), followed by sonication and centrifugation, after which the supernatant was collected and stored at −80 °C. For the separation of cytoplasmic and nuclear extracts, retinas were homogenized in lysis buffer (20 mM HEPES, 1 mM MgCl_2_, 10.8% sucrose, 50 mM β-mercaptoethanol, 1% protease inhibitor cocktail [Sigma-Aldrich], and 0.5% Nonidet P40 [NP-40]). The lysate was centrifuged for 5 min at 1500× g, and the supernatant was separated and used as the cytosolic fraction. The pellet was washed once with lysis buffer (without NP-40) and proteins were extracted by adding 2 volumes of low-salt buffer and one volume of high-salt buffer (20 mM HEPES buffer pH 7.9, 1.5 mM MgCl_2_, 0.2 mM EDTA, 1% β-mercaptoethanol, 0.5% protease inhibitor cocktail, 25% glycerol, and 20 mM or 0.8 M KCl, respectively). For western blot analysis, protein samples (10 μg each) were boiled for 10 min and subjected to SDS–PAGE. They were then transferred to a nitrocellulose membrane (GE Healthcare, Buckinghamshire, UK), which was incubated with primary antibodies, followed by peroxidase-conjugated goat-antimouse or goat-antirabbit secondary antibodies. Signals were visualized by chemiluminescence using the Amersham ECL Western Blotting Analysis System (GE Healthcare).

## Results

### *Cerkl* expression and splice-variants in the mouse retina

**Figure 1:**
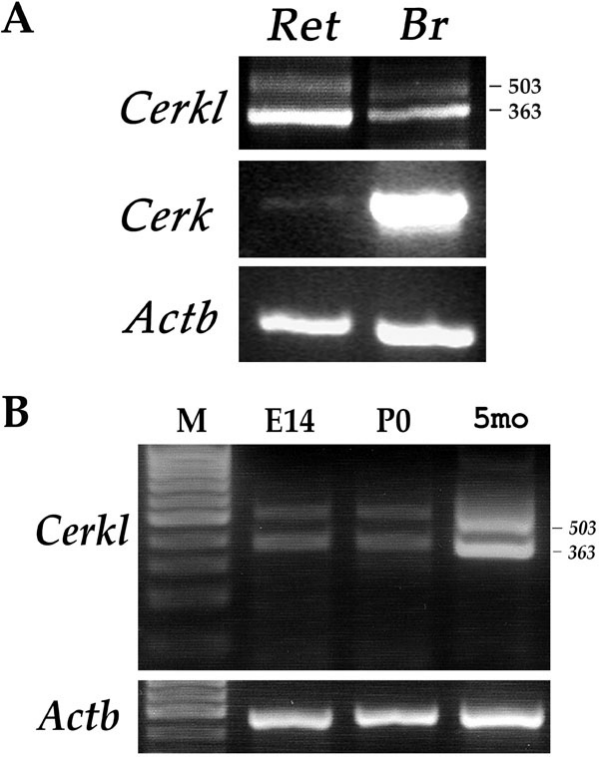
Expression analysis of *Cerkl* in the mouse eye. **A:** Reverse Transcription-Polymerase Chain Reaction (RT–PCR) analysis of *Cerk* (412 bp) and *Cerkl* expression in mouse brain (Br) and retina (Ret). *Cerkl* expression was tested using primers located in exons 1 and 2 (see Figure 2B). The two PCR products obtained for *Cerkl* (503 and 363 bp) represent different splice-isoforms. **B:** RT–PCR analysis of *Cerkl* in the mouse eye at different developmental time points: embryonic day 14 (E14), newborn (P0), and 5 months (5 mo). The analysis indicates *Cerkl* expression at all time points tested. β-actin (*Actb*; 437 bp product) served as an internal control for RNA quality and quantity. M: size marker.

RT–PCR analysis revealed that in the mouse retina, *Cerkl* is highly expressed, while expression of its homolog, *Cerk*, is relatively low (Figure 1A). To examine the ocular expression of *Cerkl* at different developmental time points, we performed RT–PCR analysis of total RNA from the mouse eye. *Cerkl* was found to be expressed in the developing mouse eye as early as embryonic day 14 (E14), although its expression rate in the embryonic and newborn (P0) eye was lower compared to that in the adult eye (Figure 1B).

**Figure 2:**
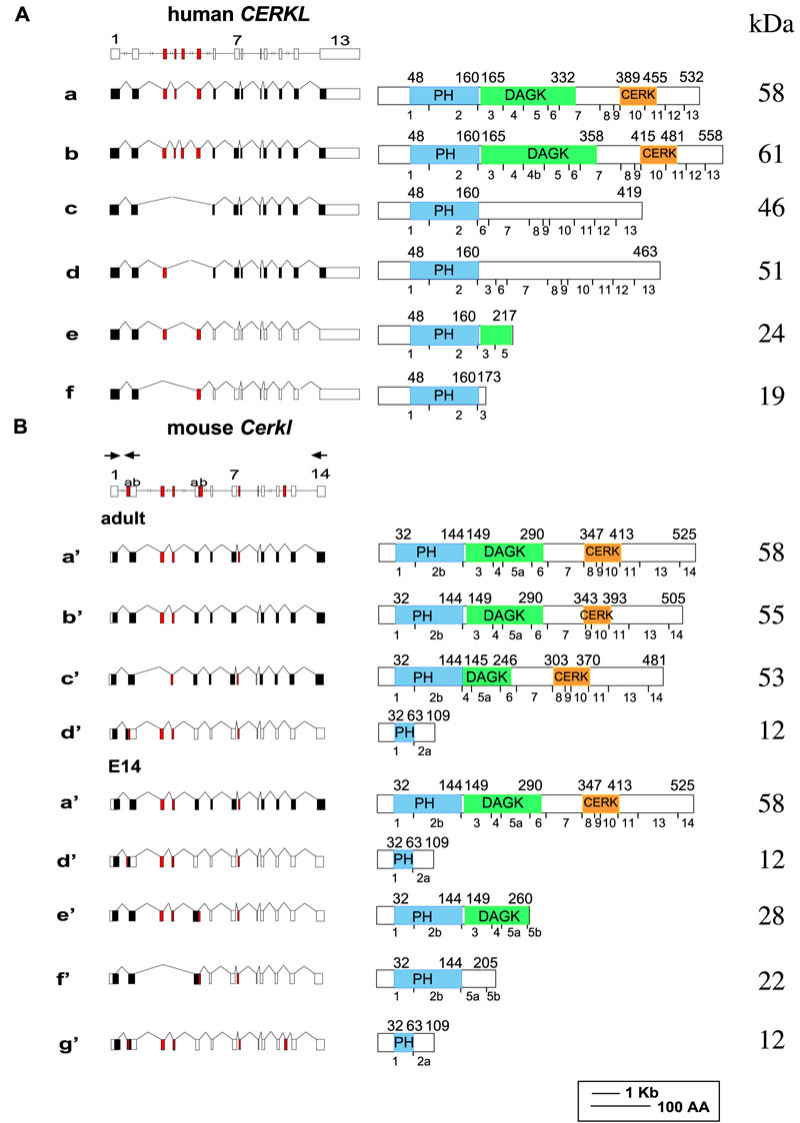
*CERKL* gene and splice-variants in human and mouse retina. A schematic representation of *CERKL* genes (drawn to scale), splice-variants (left panels), and expected protein products (right panels) is shown. In the splice-variants illustrations, filled boxes represent coding exons, and open boxes represent non-coding exons. Alternatively spliced exons are marked in red. In the protein products illustrations, the PH, DAGK, and CERK homology domains are indicated. The borders of each domain are marked by their amino acid positions, indicated above each protein. The breaks between exons are indicated below each protein. Protein molecular weight in kDa is shown on the right. **A**: Human *CERKL* gene and splice-variants previously identified in the human retina. Variants e and f correspond to AY690333 and AY690332 [13]. **B**: Mouse *Cerkl* gene and splice-variants found in the mature and embryonic (E14) mouse retina. Two of the mouse isoforms (isoforms d’ and g’) encode for the same short protein product. Mouse isoform a’, which is present in both adult and embryonic samples, is equivalent to human isoform a, and mouse isoform f’ is equivalent to human isoform f. Locations of PCR primers used for RT–PCR analysis and for splice-variant identification (forward primer located in exon 1 and reverse primers located in exons 2 and 14) are indicated by arrows above the schematic representation of the murine gene.

Comparison of the mouse *Cerkl* full-length cDNA sequence (GenBank NM_001048176) to the mouse genome sequence at the UCSC Genome Browser initially revealed that the mouse gene comprises 13 exons. The exon structure of human and mouse *CERKL* genes is highly conserved (Figure 2). Tuson et al. [13] identified four main *CERKL* splice-variants in the human retina (isoforms a-d), and two additional non in-frame variants that would generate prematurely truncated proteins (AY690333 and AY690332; Figure 2A, isoforms e and f, respectively). To identify the splice-variants of *Cerkl* in the mouse retina, we performed RT–PCR amplification of RNA from adult and embryonic (E14) retina. Four different *Cerkl* splice-isoforms were identified in the adult retina (variants a’-d’). In the embryo, five different isoforms were detected (isoforms a’, d’-g’). One of the embryonic isoforms (isoform g’) included a novel exon of 156 bp (exon 12). Therefore, the total number of exons in the mouse *Cerkl* gene is 14, as in the human ortholog (Figure 2B).

Three of the identified mouse retinal isoforms (isoforms a’-c’) encode for relatively long protein products including the PH domain, and the DAGK and CERK homology domains. The other isoforms encode for very short protein products, which include only a full or a partial PH domain (Figure 2B). Two of the isoforms (isoforms d’ and g’) differ by the absence or presence of exon 12. However, they both harbor exon 2a, which creates a frameshift and early termination of translation; therefore, both encode for the same short protein product of 109 AA (Figure 2B). Only two isoforms (isoforms a’ and d’) were present in both adult and embryonic samples. Comparison of human *CERKL* retinal splice-isoforms reported by Tuson et al. [13] to mouse *Cerkl* retinal splice-isoforms identified in this study indicated that only two are equivalent. Mouse isoform a’ is equivalent to human isoform a, and mouse isoform f’ is equivalent to human isoform f (AY690332; Figure 2).

### Generation and evaluation of an anti-CERKL antibody

**Figure 3:**
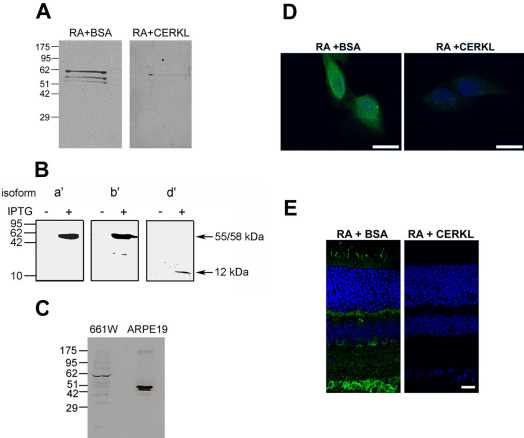
Verification of the specificity of the anti-CERKL antibody RA. **A**: Western blot analysis of mouse retinal extract with the affinity-purified RA antibody. The antibody detects a main specific band, which corresponds to the expected size of the primary and most abundant CERKL isoform in the adult mouse retina (isoform a’, 58 kDa). Two additional fainter bands slightly higher than 51 kDa, corresponding to isoforms b’ and c’ (55 and 53 kDa, respectively), can also be observed. All three bands are completely absent following pre-absorption of the RA antibody with the recombinant CERKL protein (right panel), but not with a non-specific protein (BSA; left panel). **B**: Western blot analysis of extracts from bacteria transformed with mouse *Cerkl* retinal isoforms a’ (58 kDa), b’ (55 kDa), and d’ (12 kDa). The RA antibody detects proteins of the expected sizes in IPTG-induced, but not in un-induced bacterial extracts. **C**: Western blot analysis of protein extracts from the ARPE-19 and 661W cell lines. In the mouse-derived cell line, 661W, a major band of approximately 58 kDa, which corresponds to mouse CERKL isoform a’ is detected. In the human-derived cell line, ARPE-19, two bands corresponding to human CERKL isoforms c and d (46 and 51 kDa, respectively) are detected. **D**: Immunostaining of ARPE-19 cells is omitted following pre-absorption of the RA antibody (green) with the recombinant CERKL protein (right panel), but not with a non-specific protein (BSA; left panel). Nuclei are stained with TO-PRO-3 (blue). Scale bar, 20 µm. **E**: Immunostaining of a mouse retina section is omitted following pre-absorption of the RA antibody (green) with the recombinant CERKL protein (right panel), but not with a non-specific protein (BSA; left panel). Nuclei are stained with TO-PRO-3 (blue). Scale bar, 20 µm.

To study the expression pattern and function of CERKL, we developed a specific anti-CERKL antibody (RA). The antibody was raised in rabbit against a mixture of two short peptides (15 AA each). The specificity of the RA antibody was initially tested using western blot analysis. The antibody detected a main specific band that corresponds to the expected size of the primary and most abundant CERKL isoform in the adult mouse retina (isoform a’, 58 kDa). Two additional fainter bands slightly higher than 51 kDa could also be observed, which may correspond to isoforms b’ and c’ (55 and 53 kDa, respectively). All three bands were completely omitted following pre-absorption of the RA antibody with the recombinant CERKL protein, but not with a non-specific protein (BSA; Figure 3A).

To further verify the specificity of the RA antibody, we expressed three CERKL mouse retinal isoforms (isoforms a’, b’, and d’) as recombinant proteins in bacteria under an IPTG-inducible promoter. Western blot analysis with the RA antibody detected proteins of the expected sizes in induced, but not in un-induced bacterial extracts (Figure 3B).

### Subcellular localization of CERKL in cultured cells

**Figure 4:**
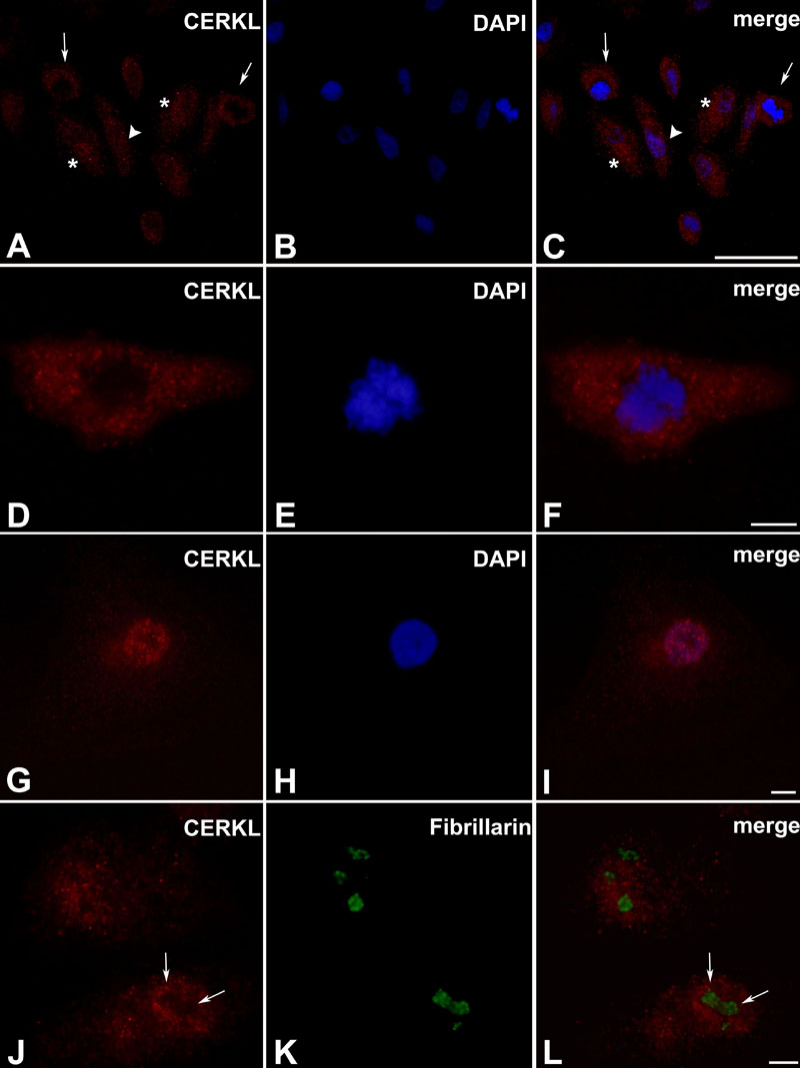
CERKL subcellular localization in ARPE-19 cells. Cells were stained with the RA anti-CERKL antibody (red) and with DAPI for nuclear staining (blue). **A**-**C**: The subcellular localization of CERKL is variable. It can be located in both the nucleus and the cytoplasm (arrowhead), or only in the cytoplasm (arrows). In many cells, CERKL is concentrated in the perinuclear region (asterisks). Scale bar, 50 µm. **D**-**F**: A cell in which CERKL is absent from the nucleus. Scale bar, 10 µm. **G**-**I**: A cell in which CERKL is concentrated in the nucleus. Scale bar, 10 µm. **J**-**L**: CERKL is absent from nucleoli (arrows), which are stained by the nucleolar marker Fibrillarin (green). Scale bar, 10 µm.

To date, the subcellular localization of CERKL was tested by overexpression of the tagged protein in various cell lines that were not of retinal origin [11,13]. Therefore, we used the anti-CERKL antibody generated in this study by our group (RA) to test the subcellular localization of the endogenous CERKL in two cell lines: ARPE-19 (derived from human retinal pigmented epithelium [RPE]) [17] and 661W (derived from mouse photoreceptor cells) [18]. We initially performed western blot analysis of protein extracts from these two cell lines with the RA antibody (Figure 3C). In the mouse-derived cell line, 661W, a major band of approximately 58 kDa corresponding to mouse CERKL isoform a’ (Figure 2B) was detected; whereas, in the human-derived cell line, ARPE-19, two bands corresponding to human CERKL isoforms c and d (46 and 51 kDa, respectively) (Figure 2A) were detected. Immunostaining of ARPE-19 cells with the anti-CERKL antibody revealed that the subcellular localization of CERKL is variable (Figure 4). CERKL is diffusely distributed in the cytoplasm, and in many cells, it is highly concentrated in the perinuclear region (Figure 4A-C). In most cells, CERKL is also present in the nucleus (Figure 4A-C,G-L); however, in a few cells in each field, CERKL was completely absent from the nucleus (Figure 4A-F). We hypothesized that the localization of CERKL to the nucleus may be correlated to the cell cycle state, but no such correlation was found (data not shown). Staining of ARPE-19 cells was eliminated following pre-absorption of the RA antibody with the recombinant CERKL protein, further indicating the specificity of this antibody (Figure 3D).

**Figure 5:**
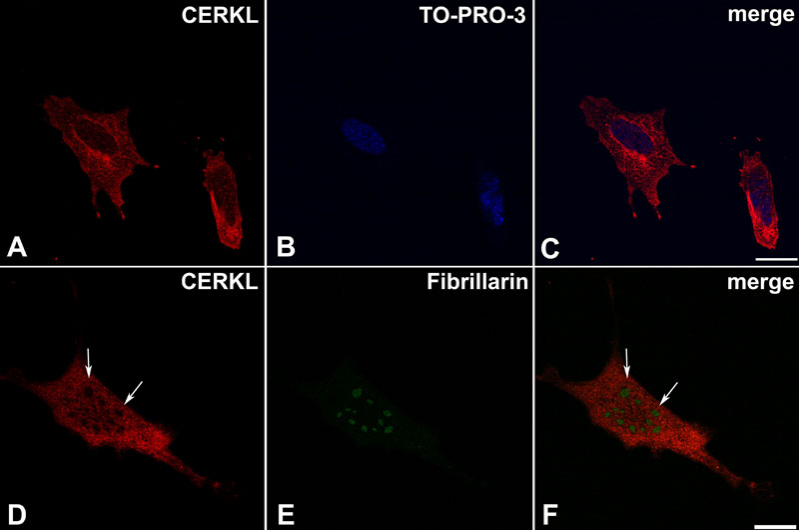
CERKL subcellular localization in 661W cells. Cells were stained with the RA anti-CERKL antibody (red) and with TO-PRO-3 for nuclear staining (blue). **A**-**C**: CERKL can be located in both the nucleus and the cytoplasm, where it is concentrated in the perinuclear region. Scale bar, 20 µm. **D**-**F**: CERKL is absent from nucleoli (arrows), which are stained by the nucleolar marker Fibrillarin (green). Scale bar, 20 µm.

The subcellular localization of CERKL in 661W cells was more uniform. In most cells, CERKL was distributed in both the cytoplasm and the nucleus, and highly concentrated in the perinuclear region (Figure 5A-C). Interestingly, while in previous studies the exogenously expressed CERKL was found to be concentrated in nucleoli [11], in both ARPE-19 and 661W cells, endogenous CERKL was consistently absent from nucleoli (Figure 4J-L and Figure 5D-F). This finding could be explained by cell type differences. Alternatively, it is possible that the previously observed nucleolar localization was an artifact of overexpression, which does not reflect the localization of CERKL at physiologic levels.

### Localization of CERKL in the mouse retina

**Figure 6:**
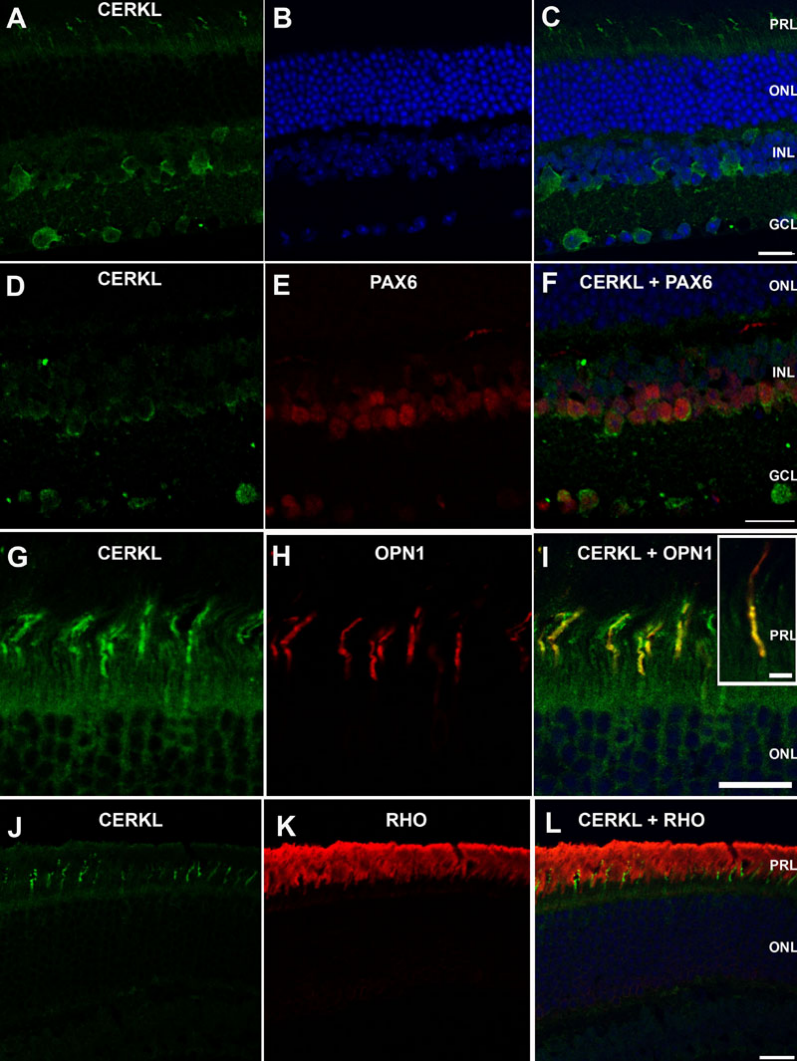
CERKL expression pattern in the mouse retina. Serial sagittal sections of adult mouse retina were immunostained with the RA anti-CERKL antibody. **A**-**C**: CERKL staining (green) shows expression in the photoreceptor cell layer (PRL), the inner nuclear layer (INL), and the ganglion cell layer (GCL). **D**-**F**: Double staining for CERKL (green) and PAX6 (red), a marker for amacrine cells, in the INL and GCL. **G**-**I**: Double staining for CERKL (green) and OPN1 (red), a marker for cone photoreceptor cells. The insert in panel **I** shows a higher magnification of a double-stained cone photoreceptor. **J**-**L**: Double staining for CERKL (green) and RHO (rhodopsin; red), a marker for rod photoreceptor cells. Nuclei are stained with TO-PRO-3 (blue). Note that CERKL is highly expressed in cone photoreceptors, while its expression level in rod photoreceptors is very low. No staining was observed when sections were stained with serum from pre-immune rabbits or with secondary antibody only (data not shown). PRL, photoreceptor layer; ONL, outer nuclear layer; INL, inner nuclear layer; GCL, ganglion cell layer. Scale bars, 20 µm.

Immunostaining of adult mouse retinas with the RA anti-CERKL antibody indicated that CERKL was located in the GCL, INL, and PRL (Figure 6A-C). No staining was observed in the RPE (data not shown). These results are in agreement with previously published data based on in situ hybridization [5]. However, the use of a specific antibody enabled a more detailed analysis in the present investigation. Within the INL, CERKL was located in amacrine cells, as confirmed by its co-localization with the amacrine cell marker PAX6 (Figure 6D-F). Interestingly, in the PRL, CERKL was highly expressed in the outer segments of cone photoreceptors, as indicated by its co-localization with OPN1, a cone-specific marker [19] (Figure 6G-I). In contrast, the expression level of CERKL in rod photoreceptors was very low (Figure 6J-L). Staining was completely absent following pre-absorption of the RA antibody with the recombinant CERKL protein (Figure 3E).

**Figure 7:**
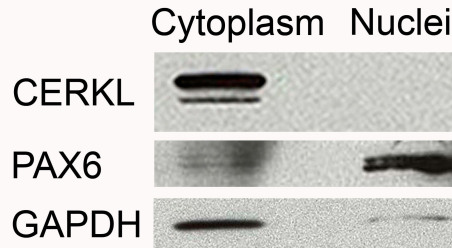
CERKL expression in the cytoplasm and nuclei of adult mouse retina. Western blot analysis was conducted on nuclear and cytoplasmic fractions of adult mouse retinas with an anti-CERKL antibody. PAX6 served as a positive control for the nuclear fraction. GAPDH served as a positive control for the cytoplasmic fraction.

In CERKL-positive mouse retinal cell types in situ, CERKL was mostly located in the cytoplasm. In photoreceptors and amacrine cells, CERKL was exclusively located in the cytoplasm and nuclear staining was not observed in these cell types (Figure 6D-L). In ganglion cells, CERKL was mostly located in the cytoplasm; however, nuclear staining was observed in a few ganglion cells (Figure 6D-F). These results were obtained in both light- and dark-adapted retinas. To further verify this finding, we performed western blot analysis of cytoplasmic versus nuclear fractions derived from mouse retinas. CERKL was exclusively detected in the cytoplasmic fraction (Figure 7).

## Discussion

*CERKL* is a novel gene of unknown function. It was named *CERKL* due to its homology to *CERK* [11]. Our analysis revealed that in the mouse retina, *Cerkl* is highly expressed, but *Cerk* expression is very low (Figure 1A). Based on this finding, it may be hypothesized that in the retina, CERKL replaces CERK. However, to date, there is no evidence that these two proteins actually share a similar function. Unlike CERK, CERKL does not phosphorylate ceramide or any other lipid substrate in vitro [11,13]. Moreover, in the retinas of *Cerk*-deficient mice, ceramide levels were increased and ceramide-1-phosphate (C1P) levels were decreased. In contrast, wildtype levels of both ceramide and C1P were found in the retina of *Cerkl* null mice [14]. These findings indicate that despite its relatively low expression level in the retina, CERK, and not CERKL, is responsible for ceramide phosphorylation in this tissue [14].

The high expression level of *Cerkl* in the retina, in addition to the severe retinal phenotype associated with human *CERKL* mutations, indicate that this gene plays a crucial role in retinal activity. Moreover, we found that *Cerkl* is expressed in the mouse eye as early as E14 (Figure 1B), which suggests that *Cerkl* may be important for retinal development, in addition to its role in the mature retina. Tuson et al. [13] reported that the overexpression of CERKL prevented cells from entering apoptosis induced by oxidative stress conditions, which implies a protective role for CERKL in photoreceptors. This possibility requires further investigation.

*CERKL* transcripts are alternatively spliced in both human and mouse retinas. Tuson et al. [13] identified four main *CERKL* splice-variants in the human retina (isoforms a-d), and two additional non in-frame variants that would generate prematurely truncated proteins (isoforms e and f). They considered these two variants to be aberrant transcripts. Since the human exon 4b, which is included only in isoform b, has no equivalent in the mouse gene (Figure 2), they speculated that in the mouse retina, there are only three *Cerkl* splice-isoforms [13]. However, we identified a total of seven distinct *Cerkl* splice-variants in the mouse retina. Mouse variant a’, which encodes for the longest protein product of 525 AA, was present in the human retina as well (variant a). Interestingly, the only other variant common to both human and mouse was mouse isoform f’, which is equivalent to human isoform f (AY690332; Figure 2). The presence of this short isoform in both human and mouse retinas indicates that it is probably not an aberrant product, and may have biologic significance. For example, it may have a regulatory role at the RNA or protein level.

Our findings regarding the expression pattern of CERKL within the mouse retina are noteworthy. Previously, based on in situ hybridization, *Cerkl* was reported to be expressed in photoreceptors in general [5], but our analysis indicates that in the murine retina, CERKL is highly expressed in cone photoreceptors, with a very low expression level in rods (Figure 6). This finding correlates with the CERKL-associated phenotype in humans, which is characterized by a significant involvement of cones, and is often diagnosed as CRD [6,8]. Nevertheless, based on this expression pattern, it is difficult to explain rod degeneration in humans with *CERKL* mutations. It is possible that in the human retina, the expression level of CERKL in rods is higher than in the murine retina. Alternatively, despite its low expression level, CERKL may still be crucial for rod survival. A third option is that rod degeneration may be secondary to cone degeneration. This option is less likely, as at least in some patients with *CERKL* mutations, a similar degree of rod and cone dysfunction is observed [8].

Within cone photoreceptors, CERKL was found in the outer segments, which are highly specialized organelles consisting of a series of discrete membranous discs that are densely packed with rhodopsin and other proteins involved in the phototransduction cascade. Moreover, they lack most of the proteins involved in other cellular functions [20]. It may be hypothesized that CERKL’s function is directly linked to the phototransduction mechanism; however, this possibility requires further investigation.

Another important finding from our analysis is related to the intracellular localization of CERKL, and specifically to its presence in the nucleus. Two putative NLS and NES were identified in the CERKL protein, and these appear to be responsible for the trafficking of CERKL between the cytoplasm and the nucleus in cultured cells [12,15]. CERKL was detected in the nucleus in several cell lines, including retina-derived cell lines of both human and mouse origin (Figure 4 and Figure 5). Moreover, a missense mutation that alters NLS2 was identified in members of a Pakistani family affected with retinal degeneration [7], and it was suggested that a defective nucleocytoplasmic shuttling mechanism might be responsible for retinal degeneration in CERKL mutants [12]. Our analysis indicated that in cultured cells, there is intercellular variability regarding the localization of CERKL. In ARPE-19 cells, CERKL was located in the nucleus in some, but not all cells within the same culture (Figure 4), a finding made previously [11–13,15]. The reason for this intercellular variability and the signals that affect the nuclear localization of CERKL are currently unknown. Furthermore, we found that CERKL is almost exclusively localized to the cytoplasm in the murine retina in situ, following both dark- and light- adaptation (Figure 6 and Figure 7). Whether CERKL is actually transported to the nucleus of retinal cells in vivo under certain conditions (e.g., following ultraviolet (UV) radiation or oxidative stress) and the functional significance of the NLS remain to be discovered.
